# Unleashing endogenous regeneration by senolytics

**DOI:** 10.1186/s12967-025-07398-y

**Published:** 2025-11-25

**Authors:** Navneet K. Boddu, Santosh Kesari, Brandy A. Chavez, Anil Bajnath, Maxim D. Wheatley, Qianyi Zhang, Yiqiao Wang, Joseph Ellis, Emma Lin, Jorge Genovese, Maria Fatima N. Robles

**Affiliations:** 1 Advanced Pain and Regenerative Specialists, Oceanside, CA USA; 2 Ashtra Health, Santa Monica, CA USA; 3 Lundquist Institute, Torrance, CA USA; 4 Institute for Human Optimization, Hannover, MD USA; 5 Department of Medicine, George Washington University, Washington, DC USA; 6 JetPatient Inc, Los Angeles, CA USA; 7 Robles BioCeutics, San Diego, CA USA; 8 Innovigen Inc, San Diego, CA USA

## Abstract

Regenerative medicine holds significant promise for the treatment of chronic diseases by harnessing the body’s innate capacity to repair and restore physiological function. However, regenerative activity declines markedly with age, a phenomenon traditionally attributed to depletion/exhaustion of progenitor cell pools. We propose an alternative, complementary mechanism: that age-associated accumulation of senescent cells contributes to impaired regeneration through the secretion of “anti-regenerative” factors. Senolytic therapies, which selectively eliminate senescent cells, may therefore exert therapeutic effects in part by de-repressing endogenous regenerative pathways. If senolytics restore progenitor cell function, their use in combination with interventions known to stimulate endogenous progenitor activity, yet not fully optimized for clinical translation, may provide a powerful strategy to enhance tissue repair. In this review, we examine current research on cellular senescence and the therapeutic potential of senolytics across diverse chronic disease contexts. We summarize the presence and role of endogenous regenerative cells in multiple organ systems and highlight mechanisms by which senescent cell-derived secretory factors inhibit regeneration. Finally, we propose potential applications in which senolytic therapy could be leveraged to augment both homeostatic regeneration and regeneration stimulated by therapeutic interventions.

## Introduction

Unlike aging, a progressive and continuous process throughout life, senescence is a dynamic and variable biological circuit. It adapts to different vital conditions, including pathological processes and their possible resolution, and extends from embryogenesis to the last events associated with aging [1]. The actions of senescence cells are wide and diverse, involved in different and sometimes opposite processes, and mediated for complex molecular systems that can regulate vital biological processes, many of which are non-related to aging events [2, 3]. Modulating the amount of senescence cells and their secretory activity offers a method for preventing age-related conditions and extending life expectancy.

Cardinal features of senescent cells include morphological and metabolic changes, chromatin reorganization, and altered gene expression [4]. Importantly, these cells can alter their composition in response to specific biological conditions. The physiological context determines whether senescent cells exert therapeutic or pathological effects. For example, the process of senescence can prevent the malignant transformation of damaged cells by blocking proliferative ability after damage has occurred. However, the onset of senescence may contribute to many age-related diseases, such as cancer, and tissue degeneration by secretion of inflammatory mediators. Interestingly, an increase in senescent cells is not necessarily associated with negative aging processes. Their increase over time benefits the healing of wounds that occur more frequently in older adults [5]. Likewise, replication changes inherent to senescent DNA affect the replication of damaged DNA, representing an important natural antitumor mechanism [6, 7]. However, the harmful effects of age on renal, cardiovascular, and neurological pathology primarily associated with SASP are undeniable. The fundamental importance of senescence in disease pathology is illustrated by the fact that significant improvements in a wide range of non-related disease conditions have been described in response to treatment with senolytics. In cancer, studies show that tumor cells induce the generation senescent cells in the tumor microenvironment. This has been correlated with poor survival in various tumors [8], as well as resistance to chemotherapy and radiation therapy [9–12]. In type 2 diabetes secondary complications such as neurocognitive defects [13], kidney failure [14, 15], and retinopathy [16], have been reduced by senolytics. Additionally, in ischemia/reperfusion (IR) injury, recovery from both cerebral [17–19], and cardiac injury [20, 21], is accelerated by senolytics.

Among the most notable changes in a senescent cell is its ability to alter gene expression and interact with various systems of the body through a proinflammatory phenotype defined as Senescence-Associated Secretory Phenotype (SASP). SASP have different and, in some instances, critical biological impacts depending on the context in which it is generated. Thus, SASP may be involved in the early elimination of transformed cells, induce persistent inflammatory processes, or actively participate in the aging process and associated diseases, such as cancer, diabetes, and chronic inflammation [22].

It is known that the production and secretion of SASP factors result in local inflammation and immune cell recruitment [23]. These cells, like macrophages, neutrophils, and lymphocytes, can eliminate the SASP signal cells produced, but they can interact with other immune cells, which inhibit these cell toxic effects. Through their SASP, cells can affect surrounding cells and tissues by remotely inducing inflammation, attracting immune cells, spreading senescence to other cells, altering stem and progenitor cell function, and impacting endocrine and metabolic function [24, 25]. SASP varies among senescent cells depending on the cell type from which they originated, how they were induced, the time elapsed since induction, and the modulated molecular mediators. The most frequently observed SASP peptides and proteins include cytokines IL6, IL8, IL1β, and GMCSF; chemokines MCP1 to 4, MIP1α (MIP3α), GROα, RANTES, RARRES2, matrix proteases and their inhibitors (MMP 1, 3, 9, and 12 and TIMP), and factors that modulate stem cell function, tissue development, and angiogenesis, including activin A and TGF-β [22, 26]. The bioactive elements of SASP also consist of microvesicles and exosomes, microRNAs along with other non-coding RNAs, fragments of mitochondrial DNA and various nucleotides, reactive oxygen species (ROS), prostanoids, ceramides, bradykinins, protein aggregates, and additional factors that may also promote senescence and intensify inflammation [27]. This data confirms that the effects of senescent cells and the immune system, including SASP production, are highly variable depending on the original cell population age and environmental conditions.

## Senescent cells produce accelerated degeneration

Because of SASP components, neighboring cells can undergo senescence in both an autocrine and paracrine manner, suggesting that senescence may trigger an inflammatory microenvironment that can eliminate senescent cells, or in some cases promote senescence in neighboring cells [28]. Cellular senescence plays a dual role in both development and tissue repair. On one hand, it aids in clearing cellular debris, reduces fibrosis, induces epigenetic changes, and acts as a strong barrier against tumor formation. On the other hand, it can encourage the spread of senescence, lead to DNA damage, cause additional tissue injury, and ultimately contribute to tissue deterioration and various age-related diseases. The role of the SASP in tissue repair appears to be extremely important. Furthermore, beyond the direct effect on cell division and clearance, the SASP is a key mechanism by which senescent cells influence injury. The SASP acts as a proinflammatory response that activates and reinforces the senescent phenotype in surrounding cells, mediating the spread of senescence throughout the tissue, known as “bystander senescence” [29].

Paracrine senescence, for example, intensifies biliary injury and impairs liver regeneration. Ablation of SASP, through inhibition of TGFβ, increases hepatocyte proliferation, decreases fibrosis, and improves overall liver function [30]. Senescent cells, through the secretion of the SASP factor PDGF-AA, play a significant role in wound healing by stimulating myofibroblast differentiation and promoting granulation tissue formation [5]. As mentioned earlier, the effects of SASP local secretion depend on how long it is present and active. This time-dependent effect appears to be directly related to SASP components, their interaction with immune signals and cells, and the possibility of regulating local and regional inflammatory activity. Prominent inflammatory components and adverse consequences occur primarily when SASP is long-lived, and it may be beneficial when transient, acting as a key component of early tissue remodeling [31–33].

## Pathological impact of senescent cells

A number of pathologies can be caused by senescence cells and their SASP in different organs and systems. There are many of these disorders that can be improved by the use of senolytics and/or senomorphics. Below is a non-exhaustive list of conditions in which senolytics have provided therapeutic impact.

### Cardiovascular conditions

Senescent cell accumulation, and the production and release of SASP components have been associated with age-related cardiac pathologies such as heart failure, myocardial ischemia and infarction, and cancer chemotherapy-related cardiotoxicity [34]. The small molecule bcl-2 family inhibiting senolytic Navitoclax improved angiotensin 2 induced cardiac dysfunction by augmenting the left ventricular ejection fraction [35] while in ischemia reperfusion model senolytics also improved recovery [36]. Additionally, senescent cells contribute to endothelial dysfunction and are found in advanced atherosclerotic lesions promoting disease progression. The use of senolytic agents can prevent atherogenesis and improve endothelial function [37]. The use of senolytics like Dasatinib and Quercetin alleviates endothelial cells senescence [38].

### Neurological conditions

The accumulation of senescence cells in the nervous system is a natural phenomenon in aging organisms. This increase is evident in the neural and glial population in different areas of the central and peripheral nervous system. The number of senescent cells is greatly increased in individuals with degenerative diseases. Senescence cells can be detected even in the population of Neural Precursor Cells (NPC). These senescent NPCs show a reduced proliferative capacity with impact in neurogenesis and cognitive function [39]. Senescence cells are clearly involved in neural pathologies like Alzheimer’s disease, Parkinson disease and Amyotrophic Lateral Sclerosis [40]. Interestingly, brain trauma results in an increase in senescence cells which may contribute to sequalae. The application of senolytics such as Dasatanib and Quercetin showed an significant improvement in memory and cognitive abilities [41, 42]. Senolytics have increasingly been considered as potential treatments for nervous system degenerative disorders, with special consideration being given to their ability to cross the blood-brain barrier [43].

### Cancer

Induction of senescence has been proposed as a cancer-suppressing mechanism, but senescence cells induce oncogenesis by promoting chronic inflammation and immune suppression through SASP components [44]. The SASP acts as an immunosuppressive agent in the advanced stage of cancer, promoting tumor growth. As a result of these findings, senolytics and senomorphics may have a relevant therapeutic role in the suppression of cancer by selective elimination of senescent cells [45]. Fisetin, a potent senolytic, has been studied in a variety of cancers to determine its efficacy alone or in combination with other anticancer agents [46–51]. Fisetin treatment inhibits cancer cell proliferation and is proapoptotic in vitro and in mice, and suppresses inflammation, migration, and metastasis [52]. In parallel, various senomorphic drugs are under study, including metformin and inhibitors of mTor. Preliminary results show that they can inhibit some components of the SASP avoiding the effects mentioned above. Several drugs with senolytic or senomorphic activity are being studied as potential anticancer treatments [45].

### Skeletal pathology

Osteopenia and osteoporosis affect the elderly in a large number of ways with a high impact on survival [53]. According to the findings demonstrating that targeting senescent cells reduces bone resorption without a concomitant reduction in bone formation [90] and reduces frailty [88], intermittent administration of senolytics can slow bone loss, reduce fracture risk, and extend health span in humans [53–55]. The regular use of senolytics and senomorphics in skeletal pathology is still far from a general clinical application across different pathologies and trauma.

### Other conditions

As well as the above conditions, there are many other pathologies in which the involvement of senescent cells has been studied. Diabetes mellitus [56], infertility [57, 58], adrenal gland dysfunction [59], and hypothyroidism are some of them [60]. In these multiple conditions, it is expected the utility of senolytics and senomorphics treatments, opening a new area of therapy for many diseases. Nevertheless, it is already necessary to define drug, dose, time, and benefits for reducing senescence population or SASP for each case, as well as discard any potential adverse effects or biological negative consequences.

### Removal of senescent cells stimulates regeneration

Historically it has been perceived that senescent cells play a passive biological role, however, as reviewed above, senescent cells through production of SASP and other mechanisms cause active tissue injury and in some cases stimulate the process of “infectious senescence”. Here we propose that senescent cells restrain regenerative potential of organisms and that by reducing senescent cell burden and/or effects we can enhance the ability of aged individuals to regenerate at a cellular and organ level.

## Endogenous regenerative mechanisms

There are several mechanisms by which the body regenerates after injury. In some situations, regeneration happens because of progenitor cells exiting the bone marrow and migrating to the injured area, in other situations local tissue stem cells are activated by injury and differentiate to generate replacement cells.

### Endogenous regeneration after stroke

In stroke patients, bone marrow stem cells (BMSCs) play a significant role in recovery. After a stroke, the brain experiences ischemic damage, triggering an inflammatory response that signals the bone marrow to release stem cells, primarily endothelial progenitor cells (EPC), hematopoietic and mesenchymal stem cells (MSC), into the bloodstream [61–63]. These cells are drawn to the site of injury by chemotactic signals, such as stromal cell-derived factor-1 (SDF-1), which is upregulated in the ischemic brain [64]. Once at the infarct site, BMSCs contribute to repair by differentiating into neural-like cells or supporting tissue regeneration through paracrine effects. This natural mobilization is a critical step, as it harnesses the body’s innate repair system without requiring invasive interventions, potentially improving outcomes in the acute and subacute phases of stroke. The therapeutic potential of BMSCs lies in their multifaceted contributions to brain repair post-stroke. Mesenchymal stem cells, for instance, secrete neurotrophic factors like brain-derived neurotrophic factor (BDNF) and vascular endothelial growth factor (VEGF) [65], which promote neurogenesis, angiogenesis, and synaptic plasticity. These factors create a regenerative microenvironment that supports the survival of neurons in the penumbra—the salvageable tissue surrounding the infarct. Additionally, BMSCs modulate the immune response by reducing pro-inflammatory cytokines and promoting anti-inflammatory pathways, which mitigates secondary damage caused by excessive inflammation. Studies in animal models have shown that this natural recruitment of BMSCs can reduce infarct volume and improve functional recovery, highlighting their role in both structural and functional restoration of the brain [66]. Thus, one method of endogenous stem cell mediated repair is mobilization of bone marrow derived stem cells into the area of injury.

In addition to bone marrow derived cells helping the brain regenerate, there are brain-endogenous cells that also contribute to brain repair. These cells, neural progenitor cells (NPCs), primarily radial glia-like cells expressing markers like Nestin and Sox2, reside in the subgranular zone (SGZ) and continuously generate new granule neurons throughout adulthood, a process known as adult neurogenesis [67]. Following brain damage, such as traumatic brain injury or ischemic stroke, NPCs in the dentate gyrus are activated by injury-induced signals, including growth factors like brain-derived neurotrophic factor (BDNF) and fibroblast growth factor-2 (FGF-2), as well as inflammatory cytokines [68].

This activation prompts NPCs to proliferate and differentiate into mature neurons and glial cells, which integrate into the hippocampal circuitry to restore cognitive functions like learning and memory. The newly formed neurons enhance synaptic plasticity, facilitating network remodeling in the damaged hippocampus, while glial cells support tissue repair by modulating inflammation and providing structural support [69]. The regenerative potential of dentate gyrus NPCs is further amplified by their paracrine effects and interaction with the post-injury microenvironment. NPCs secrete neurotrophic factors, such as vascular endothelial growth factor (VEGF) and insulin-like growth factor-1 (IGF-1), which promote angiogenesis, reduce neuronal apoptosis, and enhance the survival of newborn neurons.

### Hepatic regeneration

The best-known example of the function of endogenous stem cells is in the post-resection liver. After a portion of the liver is surgically removed or injured, the regenerative process begins with a rapid proliferative response from hepatocytes, the primary functional cells of the liver, which constitute about 70–80% of its mass. Within hours, hepatocytes exit their quiescent state (G0 phase) and enter the cell cycle, driven by a cascade of growth factors and cytokines like hepatocyte growth factor (HGF), epidermal growth factor (EGF), and interleukin-6 (IL-6) [70–72]. These signals, released by surrounding non-parenchymal cells such as Kupffer cells and hepatic stellate cells, stimulate DNA synthesis and cell division, peaking within 24–48 h in humans. This proliferative phase restores the liver’s functional mass, with the remaining lobes enlarging to compensate for the lost tissue. The process is tightly regulated to prevent excessive growth, ensuring the liver returns to its original size and function, typically within 1–2 weeks in humans, depending on the extent of the resection. The regeneration process also involves intricate remodeling of the liver’s vascular and extracellular matrix architecture to support the newly formed tissue. After the initial hepatocyte proliferation, other cell types, including sinusoidal endothelial cells and biliary epithelial cells, proliferate to restore the liver’s microarchitecture. Angiogenic factors like vascular endothelial growth factor (VEGF) promote the formation of new blood vessels, ensuring adequate blood supply to the regenerating tissue. Meanwhile, hepatic stellate cells contribute to matrix remodeling by depositing and degrading extracellular matrix components, maintaining structural integrity. This coordinated effort is guided by signaling pathways such as Wnt/β-catenin and Notch, which ensure precise cellular interactions.

### Cardiac regeneration

In addition to the liver, other organs also possess endogenous regenerative cells or “stem cells” for example in the heart. Cardiac stem cells (CSCs), also known as resident cardiac progenitor cells, are a rare population of endogenous cells within the heart, primarily identified by markers such as c-kit, Sca-1, or Isl-1 [73]. These cells reside in niches within the myocardium and are capable of self-renewal and differentiation into cardiomyocytes, endothelial cells, and smooth muscle cells. After a heart attack, the ischemic injury triggers a cascade of inflammatory signals, including cytokines like stromal cell-derived factor-1 (SDF-1) and vascular endothelial growth factor (VEGF), which activate CSCs [74, 75]. These cells proliferate and migrate to the infarcted region, where they contribute to tissue repair by differentiating into new cardiomyocytes to replace necrotic cells and forming vascular structures to restore blood flow. However, the endogenous CSC response is often limited, with only a small fraction of the damaged myocardium being regenerated naturally, necessitating strategies to enhance their activity. The contribution of CSCs to cardiac regeneration is augmented by their paracrine effects, which are critical for modulating the post-infarction environment. CSCs secrete growth factors, such as insulin-like growth factor-1 (IGF-1) and hepatocyte growth factor (HGF), which promote cardiomyocyte survival, reduce apoptosis, and stimulate angiogenesis [76, 77]. These factors also recruit additional progenitor cells and modulate the inflammatory response by shifting macrophages toward an anti-inflammatory phenotype, thus limiting scar formation. While CSCs hold immense potential, their regenerative capacity post-heart attack is constrained by factors like aging, oxidative stress, and the hostile microenvironment of the infarct zone, characterized by hypoxia and fibrosis [78].

### Pulmonary regeneration

In the lung one of the more important cell populations involved in regeneration is the type 2 alveolar epithelial cells (AEC2s) which are identified by markers such as surfactant protein C (SPC) [79], ATP-binding cassette subfamily A member 3 (ABCA3) [80], and the MHC II associated molecule CD74 [81]. Following lung injuries such as acute respiratory distress syndrome (ARDS), viral infections, or chemical damage, AEC2s are activated by signals from the injury microenvironment, including growth factors like keratinocyte growth factor (KGF) and epidermal growth factor (EGF), as well as inflammatory cytokines such as IL-6 [82]. These signals trigger AEC2s to proliferate and differentiate into type 1 alveolar epithelial cells (AEC1s), which form the thin alveolar barrier essential for gas exchange. This regenerative process restores the alveolar epithelium, re-establishing the lung’s structural integrity and function, typically within days to weeks depending on the injury’s severity. Beyond direct differentiation, AEC2s contribute to lung healing through paracrine mechanisms that modulate the post-injury environment. They secrete factors like transforming growth factor-β (TGF-β) and vascular endothelial growth factor (VEGF), which promote angiogenesis, reduce inflammation, and support the recruitment of other reparative cells, such as club cells or basal cells, depending on the lung region. However, chronic or severe injuries can impair AEC2 function, leading to aberrant repair and fibrosis, as seen in idiopathic pulmonary fibrosis. The regenerative capacity of AEC2s is also limited by factors like aging, oxidative stress, and depletion of the stem cell pool.

## Stimulation of endogenous regenerative mechanisms as a therapeutic intervention

As seen from the above examples, numerous organ systems are replenished by endogenous pools of stem cells and progenitor cells. In some situations, expansion of these pools by growth factor intervention can have some regenerative effects. Senescent cell anti-apoptotic pathways (SCAPs) may also play a role in enhancing senolytics via upregulation of pro-survival networks (27).

We previously discussed how bone marrow derived EPC and MSC contribute to endogenous stroke recovery. Preclinical studies have shown that transplantation of exogenous EPC can improve recovery after stroke [83]. Interestingly, stimulating mobilization of EPC from the bone marrow to periphery by administration of parathyroid hormone resulted in enhanced post-stroke recovery [84]. Post stroke mobilization of stem cells by G-CSF also resulted in improvements [85]. For stimulation of endogenous progenitor cells, administration of hCG was shown to induce dentate gyrus progenitor cell proliferation and help in stroke recovery [86]. Similar situations in which artificial stimulation of endogenous progenitors have been shown to result in therapeutic benefit.

In the treatment of critical limb ischemia (CLI), a severe form of peripheral artery disease characterized by reduced blood flow to the limbs, vascular endothelial growth factor (VEGF) serves as a powerful example of a growth factor that activates endogenous stem cells to achieve therapeutic effects. Administered through plasmid-based gene therapy (e.g., VEGF165), VEGF stimulates endothelial progenitor cells (EPCs) and mesenchymal stem cells (MSCs) to promote angiogenesis and tissue repair. By binding to VEGFR-1 and VEGFR-2 receptors, VEGF triggers PI3K/Akt and MAPK signaling pathways, driving proliferation, migration, and differentiation of these stem cells to form new capillaries in ischemic tissues. Clinical trials demonstrate that 60% of CLI patients receiving VEGF gene therapy show increased collateral vessel formation within three months, while 40% experience significant improvement in non-healing ulcers, reducing pain and amputation risk. These outcomes, including enhanced walking ability and tissue regeneration, underscore VEGF’s role in harnessing endogenous stem cells to restore vascular function and improve quality of life in CLI patients.

In the treatment of spinal cord injury (SCI), basic fibroblast growth factor (bFGF or FGF-2) is a notable example of a growth factor that activates endogenous stem cells to promote therapeutic effects. Administered via direct injection or biomaterial scaffolds in preclinical and early clinical studies, bFGF stimulates endogenous neural stem cells (NSCs) and progenitor cells in the spinal cord. By binding to FGFR1 receptors, bFGF activates ERK and Akt signaling pathways, enhancing NSC proliferation, migration, and differentiation into neurons and glial cells. In animal models of SCI, bFGF treatment has led to significant recovery, with studies showing up to 50% improvement in motor function scores within 8 weeks, alongside increased axonal regeneration and reduced scar formation. In a small human trial, patients receiving bFGF-infused scaffolds showed improved sensory function in 30% of cases, attributed to NSC-mediated tissue repair. These findings highlight bFGF’s potential to leverage endogenous stem cells for neural regeneration, offering hope for functional recovery in SCI patients.

In the treatment of chronic heart failure following myocardial infarction, hepatocyte growth factor (HGF) is an example of a growth factor that activates endogenous stem cells to achieve therapeutic outcomes. Delivered through gene therapy (e.g., HGF plasmid DNA) or recombinant protein in preclinical and early clinical studies, HGF stimulates endogenous cardiac stem cells (CSCs) and mesenchymal stem cells (MSCs) within the heart. By binding to the c-Met receptor, HGF activates PI3K/Akt and STAT3 signaling pathways, promoting CSC proliferation, migration, and differentiation into cardiomyocytes and vascular cells, while also enhancing MSC-mediated paracrine effects for tissue repair. In animal models of heart failure, HGF therapy improved left ventricular ejection fraction by up to 40% within 4–6 weeks and reduced scar size by 30%, indicating enhanced cardiac regeneration. In a phase I/II human trial, 50% of patients receiving intramyocardial HGF gene therapy reported improved heart function and reduced symptoms, linked to CSC-driven angiogenesis and myocardial repair. These results demonstrate HGF’s ability to harness endogenous stem cells to restore cardiac function in chronic heart failure.

## Senescent cells suppress endogenous stem cells

The niche in which stem cells exist contributes to both the self-renewal ability of stem cells, as well as the differentiation activity. For example, it is established that senescent microenvironment contributes to suppression of hematopoietic stem cell activity and that administration of senolytics can overcome age associated issues with hematopoiesis [87].

This concept, that senescent cells suppress endogenous stem cells is based on the fact that SASP molecules directly, as well as indirectly through recruitment of immune cells, cause inflammation and the inflammatory proteins in turn are responsible for suppressing stem cell activity. Numerous examples of inflammatory proteins suppressing stem cell activity are known at a basic level, suppression of hematopoiesis by inflammatory cytokines such as IL-1 beta, IFN-gamma and TNF-alpha is established [88]. But do inflammatory proteins also suppress other tissue-associated stem cells?

Interleukein-6 is a well-known SASP protein. Mice genetically engineered to produce interleukin-6 (IL-6) in their brain’s astroglia cells had 63% less new neuron growth in the hippocampal dentate gyrus, a key brain area for memory. IL-6 reduced the creation, survival, and development of new brain cells (neural progenitors), which were tracked using a marker called bromodeoxyuridine. However, the spread of these cells and the growth of other brain support cells (glia) stayed normal. This wasn’t due to the IL-6 gene harming the brain overall, as there was no neuron death or major changes in glial cell numbers or shapes [89].

Other inflammatory mediators are associated with active suppression of neurogenesis, for example, in a study by Ekdahl et al. (2003) the impact of inflammation on adult hippocampal neurogenesis in rats was assessed, using a model of status epilepticus and lipopolysaccharide (LPS)-induced inflammation. They found that LPS-induced inflammation, which activates microglia, caused an 85% reduction in new neuron formation in the dentate gyrus’s subgranular zone/granule cell layer, as measured by BrdUrd/NeuN double-labeled cells, without affecting mature neuron survival. This suppression was linked to increased microglial activation and the release of proinflammatory cytokines, specifically interleukin-1β (IL-1β), tumor necrosis factor-α (TNF-α), and interleukin-6 (IL-6), which were identified as detrimental to neurogenesis. Treatment with minocycline, an anti-inflammatory drug, reduced microglial activation and restored neurogenesis, highlighting the role of these inflammatory markers in inhibiting neural progenitor proliferation and survival [90].

In another study, anti-inflammatory drugs have been shown to overcome inflammation associated suppression of neurogenesis. For example, Monje et al. showed that the anti-inflammatory agent indomethacin restored neurogenesis after irradiation through suppression of inflammatory mediators such as IL-1 beta, TNF-alpha, and IL-6 [91]. In another study it was shown that whole brain irradiation (WBI) triggers a quick and ongoing increase in activated microglia, causing inflammation and reducing new neuron growth in the hippocampus. The drug tamoxifen, often used to make radiation more effective against brain tumors, was found to calm inflammation in both lab-grown microglia cells and a spinal cord injury model. In lab tests, irradiating mouse microglia cells (BV-2) with 2–10 Gy doses increased inflammatory markers IL-1β and TNF-α, and the medium from heavily irradiated cells (10 Gy) caused more astrocyte activation and fewer neurons. Treating these cells with tamoxifen (1 µM for 45 min) lowered the inflammation. In rat brains, WBI damaged the blood-brain barrier, caused brain swelling, activated microglia, increased astrocyte reactivity, and killed neurons in the hippocampus within days. Giving tamoxifen (5 mg/kg injection) immediately after radiation reduced these harmful effects. Overall, tamoxifen helps protect the brain from radiation damage by reducing harmful inflammation caused by IL-1β and TNF-α [92].

## Conclusion

Given the above examples, it appears that age-associated reduction in regenerative activity is associated with loss of regenerative activity. We therefore propose the utilization of senolytics as a means of enhancing endogenous regenerative activity, as well as augmenting regenerative activity of exogenously administered stem cells.
